# Does osteoporosis reduce the primary tilting stability of cementless acetabular cups?

**DOI:** 10.1186/s12891-015-0554-x

**Published:** 2015-04-21

**Authors:** Christoph von Schulze Pellengahr, Lars V von Engelhardt, Bernd Wegener, Peter E Müller, Andreas Fottner, Patrick Weber, Ole Ackermann, Matthias Lahner, Wolfram Teske

**Affiliations:** Department of Orthopedic and Trauma Surgery, Ruhr-University Bochum, St. Josef Hospital, Gudrunstrasse 56, 44791 Bochum, Germany; Faculty of Health, University of Witten/Herdecke, Alfred-Herrhausen-Str. 50, 58448 Witten, Germany; Department of Orthopedics, Trauma Surgery and Sports Medicine, Johanna Etienne Hospital, Am Hasenberg 46, 41462 Neuss, Germany; Department of Orthopaedic Surgery, University Hospital of Munich (LMU), Campus Großhadern, Marchioninistr. 15, 81377 München, Germany; Department of Orthopedic and Trauma Surgery, Klinikum Duisburg GmbH, Zu den Rehwiesen 9-11, 47055 Duisburg, Germany

**Keywords:** Cementless hip cup, Osteoporosis, Moment of tilt, Primary stability, Macerated bone

## Abstract

**Background:**

Cementless hip cups need sufficient primary tilting stability to achieve osseointegration. The aim of the study was to assess differences of the primary implant stability in osteoporotic bone and in bone with normal bone density. To assess the influence of different cup designs, two types of threaded and two types of press-fit cups were tested.

**Methods:**

The maximum tilting moment for two different cementless threaded cups and two different cementless press-fit cups was determined in macerated human hip acetabuli with reduced (n=20) and normal bone density (n=20), determined using Q-CT. The tilting moments for each cup were determined five times in the group with reduced bone density and five times in the group with normal bone density, and the respective average values were calculated.

**Results:**

The mean maximum extrusion force of the threaded cup Zintra was 5670.5 N (max. tilting moment 141.8 Nm) in bone with normal density and.5748.3 N (max. tilting moment 143.7 Nm) in osteoporotic bone. For the Hofer Imhof (HI) threaded cup it was 7681.5 N (192.0 Nm) in bone with normal density and 6828.9 N (max. tilting moment 170.7 Nm) in the group with osteoporotic bone. The mean maximum extrusion force of the macro-textured press-fit cup Metallsockel CL was 3824.6 N (max. tilting moment 95.6 Nm) in bone with normal and 2246.2 N (max. tilting moment 56.2 Nm) in osteoporotic bone. For the Monoblock it was 1303.8 N (max. tilting moment 32.6 Nm) in normal and 1317 N (max. tilting moment 32.9 Nm) in osteoporotic bone. There was no significance. A reduction of the maximum tilting moment in osteoporotic bone of the ESKA press-fit cup Metallsockel CL was noticed.

**Conclusion:**

Results on macerated bone specimens showed no statistically significant reduction of the maximum tilting moment in specimens with osteoporotic bone density compared to normal bone, neither for threaded nor for the press-fit cups. With the limitation that the results were obtained using macerated bone, we could not detect any restrictions for the clinical indication of the examined cementless cups in osteoporotic bone.

## Background

An important requirement for a firm osseointegration of cementless hip cups [1] is a sufficient stability of the implant in the acetabular bone in the postoperative phase (primary stability). Especially pronounced shear and tension stresses in the border zone between implant and bone should be avoided, as they are main causes of prothesis loosening [2].

The primary stability of cementless hip cups can be realised either in the form of a threaded cup or as a press-fit cup, with additional stabilizing components such as screws, wings or pegs if necessary. The primary stability of a hip cup implant is affected by the properties of bone (e.g. normal bone density or osteoporosis) as well as by the characteristics of the implant. Here, the material and the surface structure or coating play a vital role as well as the external shape, the thread form and an additional screw anchoring. The primary stability of the press-fit cup follows the principle of the anchoring of a stiff body in a slightly undersized elastic body. Here, axial loads lead to an improvement of the press-fit with an increase in stability [3].

For threaded cups, the external shape and the thread form, as well as the implant surface play a key role for the primary and secondary stability. Threaded cups should also create a press-fit, not through the insertion of an oversized cup, but through screwing in the acetabular cup. The achieved pretension in spherical threaded cups is lower than that achieved in conical threaded cups by a factor of approximately 2.5 [4].

Inter-individual variations of the acetabular bone quality could lead to a relatively good primary stability in harder healthy bone and a relatively bad primary stability in osteoporotic bone. Thus, the aim of this study was to compare the initial acetabular implant stability by comparing the extent of the tilting stability in both osteoporotic and normal bone. Besides the investigation of the influence of the bone quality, the influence of different cup designs was also assessed in our series by testing two types of threaded and two types of press-fit cups.

## Methods

The following cups were examined:

### Threaded cups

Zimmer Zintra: spherical external shape, spherical cone thread, rounded, variable thread pitch, corundum-blasted surface (roughness 4-5 μm), material: Ti 6Al 4 V.

**Figure 1:**
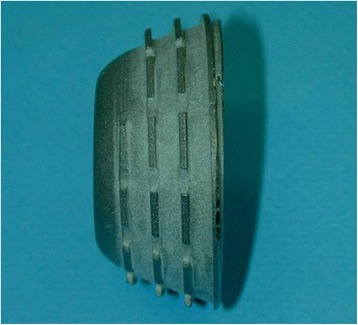
Hofer-Imhof titanium cup with a parabolic flat thread.

Smith&Nephew (formerly Intraplant) Hofer-Imhof: parabolic external shape, parabolic flat thread, parabolic overwound, corundum-blasted surface, roughness 4-6 μm, material: pure titanium (Figure 1).

### Press-fit cups

**Figure 2:**
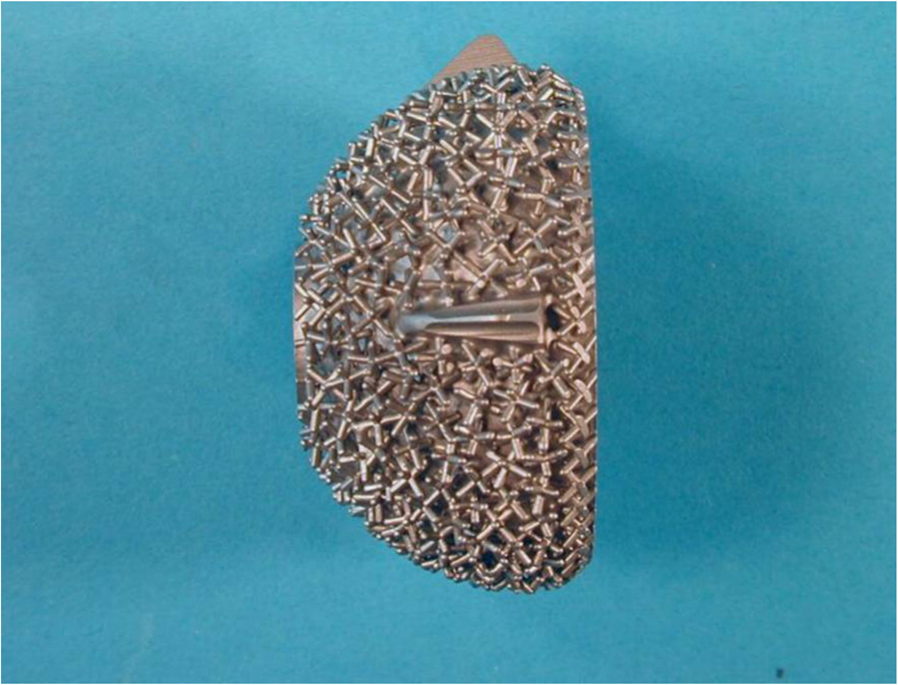
ESKA metal socket CL press-fit cup with a spherical shape, flattened pole and three-dimensional grid structure.

ESKA Metallsockel CL (engl. metal socket CL): spherical shape, flattened pole, surface: three-dimensional grid structure (tripods), cobalt-chrome-molybdenum, undersized milling, antirotational mechanism with three wings, oversizing for press-fit (1.2 mm) in an acetabulum milled to a size of 50 mm (Figure 2).

Zimmer Monoblock: elliptic external shape, surface: trabecular metal, pore size approx. 430 μm, porosity 80%, frame: tantalum, press-fit through elliptic external form, firm inlay, oversizing for press-fit (2.0 mm) in an acetabulum milled to a size of 50 mm.

Only cups that were suitable for a prepared acetabulum with an inner diameter of 50 mm were used. For threaded cups, this corresponds to an outer diameter of 50 mm plus thread flanks. Press-fit cups normally have a slightly larger outer diameter because of their aspherical frame shape or the flattening of the pole to achieve the press-fit. Accordingly, the used press-fit cups had outer diameters between 51 and 52 mm.

Pelvic halves of macerated human cadaver specimens were used for this study. Anonymized human pelvic halves were provided for research purposes by the Institute of Forensic Medicine at the University Hospital of Munich, Germany. The anonymized use of cadaver specimens was accepted by the Ethics Committee of the Ludwig-Maximilians University of Munich (UE Nr. 011-14). The selection of specimens was conducted as follows: All specimens had an inner diameter of 46 mm - 48 mm and were milled to an inner diameter of 50 mm using the implant specific milling device. All specimens which had been chosen according to their size underwent a measurement of the periacetabular bone density. The periacetabular bone density of each specimen was measured subchondrally using a quantitative computer tomography (Q-CT) in the supraacetabular bone and at the caudal, ventral and dorsal edges of the acetabulum. The advantage of QCT for the measurement of bone densities is the ability to isolate an area of interest from surrounding tissues. Providing a size-independent, three-dimensional true volumetric measurement, QCT is widely accepted as a simple and accurate bone density measurement tool [5,6].

Specimens were divided into two groups: the “normal group” contained 20 macerated pelvic halves with an above-median bone density. The “osteoporosis group” contained 20 macerated pelvic halves with a below-median bone density.

**Figure 3:**
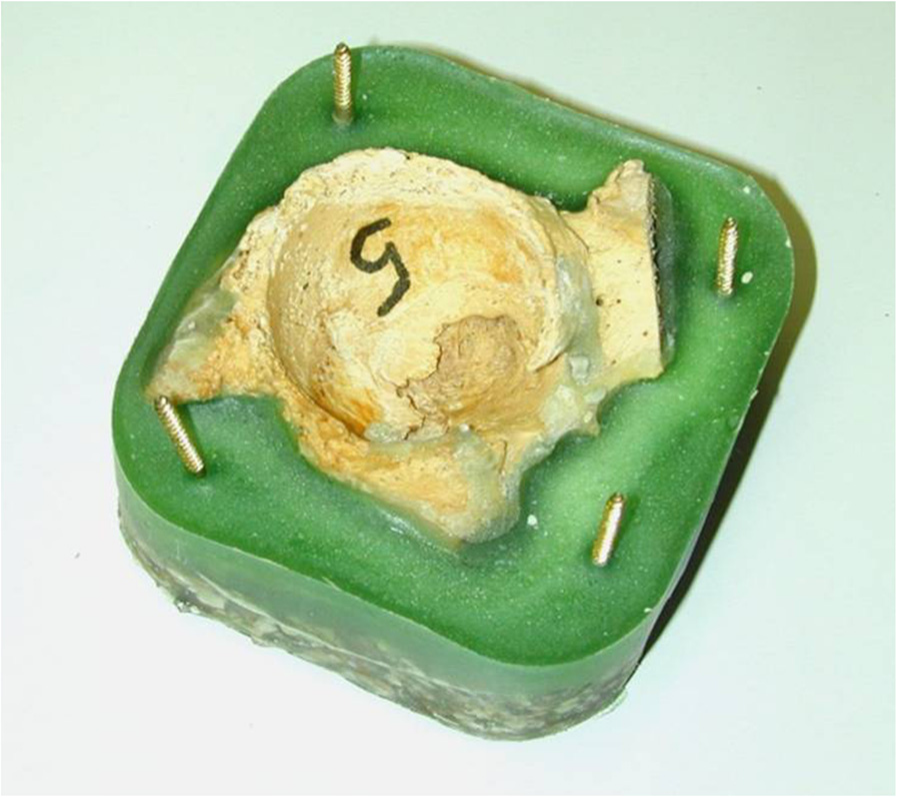
Prepared human acetabulum which is poured into methyl methacrylate.

**Figure 4:**
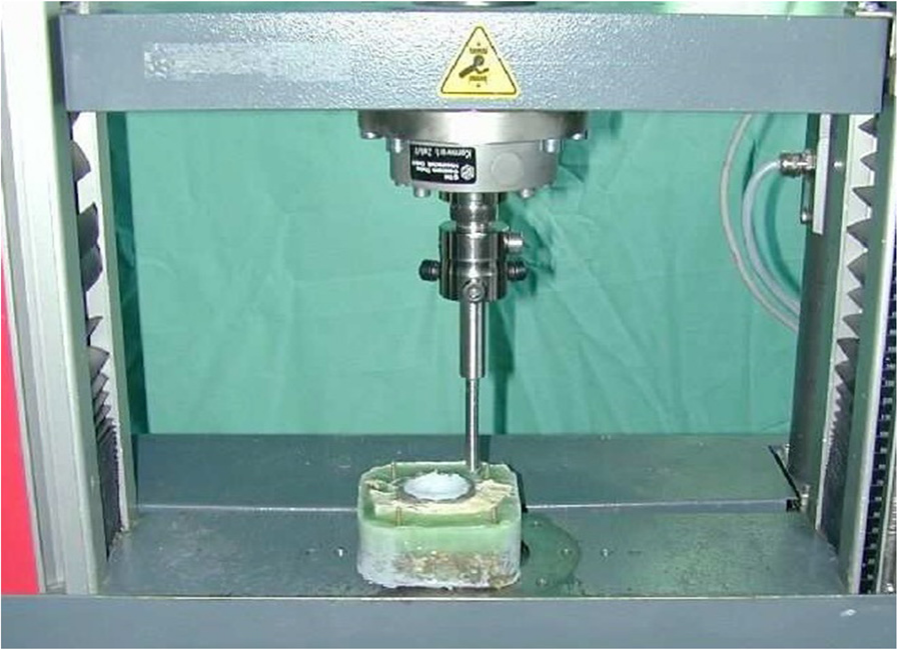
Measuring stand III (Zwick® ZO 10) to introduce the press-fit cups and to measure the extrusion forces at the edge of the acetabular cup up to the breakout.

The acetabulum was sawn out of the selected cadaver specimens, poured into methyl methacrylate to measure 10 cm × 10 cm × 5 cm (Figure 3) and clamped into the measuring stand (testing machine Zwick®) (Figure 4). 40 specimens were created in total. For each screw pan and press-fit cup, five measurements were made on specimens with normal bone density and five on osteoporotic specimens. The Zwick® ZO 10 testing machine was set to a constant forward feed. Simultaneously, the machine recorded the occurring forces.

The extrusion force and thus the tilting moments were measured up to the breakout of the examined threaded and press-fit cups. A local introduction of force was achieved with a stamp pushed down onto the edge of the cup with a constant forward feed of 0.1 mm/sec. To determine the tilting moment (M_K_), the stamp force (F_K_) was applied up to the breakout of the cup. As a loosening of prosthetic cups often occurs through a lateral tilting of the caudal cup, the application of force (F_K_) was applied at the cranial edge. Using the radius (R) of the cup, the tilting moment (M_K_) was then calculated using the following formula:

$$ {\mathrm{M}}_{\mathrm{K}} = {\mathrm{F}}_{\mathrm{K}} \times \mathrm{R} $$

(e.g. for a pan with a diameter of 50 mm: MK = FK x 0,025 m)

Confidence intervals were used to detect a significant difference between two measurements in this study. These descriptive statistics were performed under the supervision of the Department of Statistics of the Ludwig-Maximilians-University Munich. The mean, maximum, minimum, confidence interval and standard deviation were calculated for all measuring parameters. The difference between two measurements was labelled statistically significant if the accompanying 95% confidence intervals did not overlap. The statistical analysis of the measurements was performed using the program SPSS®.

## Results

In human cadavers with osteoporotic bone and normal bone density, the measurements for the threaded cup are depicted in Table 1. The differences between both groups were not significant. The differences between both types of threaded cups, the Zimmer Zintra and the Hofer-Imhof cup, which differ in shape and material of the implant, were also not significant.

**Table 1:** **Extrusion forces and tilting moments of the examined threaded cups in macerated bone with high and low bone density**

		**High bone density**		**Low bone density**	
	**Measurement**	**Max. extrusion force in N**	**Max. tilting moment in Nm**	**Max. extrusion force in N**	**Max. tilting moment in Nm**
**Zintra**	1	5829,6	145,7	5931,2	148,28
	2	8540,5	213,5	8527,7	213,1925
	3	5222,1	130,6	2383,3	59,5825
	4	4237,9	105,9	2427,1	60,6775
	5	4522,6	113,1	9472,1	236,8025
	**Mean**	**5670,5 (1720,0)**	**141,8**	**5748,3 (3315,8)**	**143,7**
**95% confidence interval**		+/- **1507,6**		**+/- 2906,4**	
**Hofer**	1	11045,8	276,1	3822,3	95,5575
	2	10778,2	269,5	10915,7	272,8925
	3	3928,0	98,2	6534,2	163,355
	4	10805,5	270,1	4290,2	107,255
	5	1850,2	46,3	8582,0	214,55
	**Mean**	**7681,5 (4437,3)**	**192,0**	**6828,9 (2972,7)**	**170,722**
**95% confidence interval**		+/- **3889,4**		+/- **2605,6**	

Table 2 shows the measurements for both types of press-fit cups, the ESKA metal socket CL and the Zimmer Monoblock, which differ in shape, material and surface of the implant. Differences between measurements in osteoporotic bone and normal bone as well as between both cup types were not significant.

**Table 2:** **Extrusion forces and tilting moments of the examined press-fit cups in macerated bone with high and low bone density**

		**High bone density**		**Low bone density**	
	**Measurement**	**Max. extrusion force in N**	**Max. tilting moment in Nm**	**Max. extrusion force in N**	**Max. tilting moment in Nm**
**Metallsockel CL**	1	3352,9	83,8	1236,9	30,9
	2	4351,9	108,8	849,2	21,2
	3	2012,1	50,3	1601,9	40,0
	4	5419,3	135,5	4599,0	115,0
	5	3986,9	99,7	2944,2	73,6
	**Mean**	**3824,6 (1260,5)**	**95,6**	**2246,2 (1533,6)**	**56,2**
**95% confidence interval**		**1104,9**		**1344,2**	
**Monoblock**	1	921,8	23,0	1584,4	39,6
	2	451,3	11,3	1283,7	32,1
	3	1241,7	31,0	702,6	17,6
	4	1683,8	42,1	measurement error	
	5	2220,6	55,5	1697,3	42,4
	**Mean**	**1303,8 (682,2)**	**32,6**	**1317,0 (445,2)**	**32,9**
**95% confidence interval**		**598,0**		**390,2**	

## Discussion

We assumed that the reduced bone density of osteoporotic acetabular bone specimens will lead to a comparatively low primary stability. According to our results, the hypothesis of this study, that the initial acetabular implant stability is different in osteoporotic and normal bone, can be rejected. Neither the threaded nor the press-fit cups in our series showed any significant reduction of the maximal tilting moments. Thus, in the clinical situation, the indication for the implantation of cementless cups examined in this study does not seem to be restricted for patients with osteoporotic bone quality.

The use of fresh frozen human hip specimens was a particular issue in this study,. First, the procurement of human hip specimens is difficult due to current legislation. Therefore, they are available on a limited basis and it is difficult to obtain a large enough number of human cadaver specimens to be able to reach a cup-specific conclusion. Furthermore, human cadaver hips differ in size and periacetabular bone density or sclerosing. Thus, the human acetabulum is more sclerotic and harder in the cranial as well as in the dorsal areas. In comparison, synthetic bone blocks such as rigid foam blocks have the same density in all directions. As a consequence of different bone densities within the acetabulum, the bone milling devices usually diverge towards the areas with lower densities. These are usually the caudal and ventral parts of the acetabulum. Additionally, the size of the acetabulum cannot be accurately assessed before extraction. As the cup implant had to have an outer diameter of 50 mm, the majority of hip specimens would have been unusable for the purposes of this study. However, according to a preliminary test, macerated human pelvic bone can be used as a valid replacement for fresh-frozen hip specimens, as the maceration of fresh-frozen human hip specimens only leads to slight and calculable changes of mechanical properties. These tests showed a maximal deviation of extrusion forces and tilting moments of 17.1% [7].

The standardized use of a 50 mm outer diameter of the cup might be a further limitation of this study. Taking into account that the tilting moment is directly dependent on the radius of the cup, we suggest that the size of the cup also plays a critical role in the primary implant stability. Thus, further trials investigating the influence of the cup size in different cup types might be of practical interest.

The moments of friction caused by daily use of a hip prosthesis lie within a range which would not have seriously threatened the tilting stability of any of the examined cups. Plitz et al. note moments of friction of between 75 and 230 Ncm (equivalent to 0.75 Nm – 2.3 Nm) in hip joint prostheses under a load of 2500 N for the material combination of cobalt-based alloys / HD-polyethylene. All other pairings tend to have lower moments of friction [8]. Larger tilting moments can occur in hip prostheses when the femoral neck of the stem has contact with the cup during extreme movements (impingement).

In a different approach, Hadjari [9] determined the tilting moments which produced micromotions of 50 micrometers in a porous-coated press-fit cup in the fresh-frozen specimen. He measured a value of 11 Nm for the examined press-fit cup, which is clearly lower than the values measured by Plitz et al. In this study, values between 152.1 Nm and >269.1 were measured in the macerated human specimens. Due to these small values, the moments of friction specified by Plitz also do not threaten osseointegration [8].

Research conducted by Ohlin found that cemented hip cups of autopsy specimens had an average maximal tilting moment of 80 Nm leading to a breakout of the cup [10]. Jäger reported on torsional moment measurements leading to a breakout of cemented cups from autopsy specimens, which lay between 457.1 cm kp (=44.84 Nm) and 1827 cm kp (=179.23 Nm), depending on the preparation of the bony acetabulum [11].

A further literature research on tilting moments of hip prosthesis cups in human bone was unsuccessful. All other authors used artificial materials as bone substitute. For six different press-fit cups in a PVC foam model, Kuhn et al. determined maximal tilting moments leading to a breakout of the implants of between 13 Nm and 23 Nm [12]. These values are far too low when compared to values measured by us on the macerated human specimen. Kuhn points out that the application of his values to the in vivo situation is difficult. The measurements made in the polyurethane block by Olory et al.[13], which lay between 7.63 NM und 55.46 Nm, should be assessed similarly. Under laboratory conditions, Wetzel et al. [14] determined maximal tilting moments of between 39.2 Nm and 50.8 Nm, which come much closer to the values on macerated human cadaver specimens. Baleani et al. [15] determined a tilting moment of 39.9 Nm for a porous coated press-fit cup in polyurethane foam up until the appearance of micromotions of 150 micrometers. The tilting moments in polyurethane foam, observed by Kody et al. [16] for a 50 mm threaded cup, measured 20 Nm, which is unrealistic in vivo.

In this study, threaded cups show a considerably higher tilting moment than the press-fit cups. The difference between conical and spherical threaded cups was relatively small. The higher tilting moment of the macro-structured press-fit cups compared to the micro-structured press-fit cups was much greater. However, the difference was not significant because of the large deviation of the macro-structured specimens.

Neither for threaded cups nor for press-fit cups did the results of this study on macerated bone specimens show any statistically significant reduction of the maximal tilting moments of osteoporotic specimens when compared to normal bone. However, a decrease of the maximal tilting moment of osteoporotic bone was indicated for the ESKA metal-socket CL press-fit cup.

If a maximal tilting moment of cemented cups of 80 Nm is used as a reference [10], it will be surpassed by all screw cups in osteoporotic and normal macerated bone. Ohlin states that micromotions of 150 micrometers occur in cemented cups with a tilting moment of 39.9 Nm [10].

The Monoblock cup was broken out by low tilting moments in over half of all cases of macerated normal bone and the Metallsockel CL press-fit cups in two cases in osteoporotic bone. Thus, it can be concluded that, especially in micro-structured press-fit cups, an impingement of the femoral neck in the early post-operative phase can lead to a breaking out of the cup or a disturbance of osseointegration.

## Conclusions

With the limitation that the results were obtained using macerated bone, there were no restrictions for the indication of the examined cementless cups in osteoporotic bone.
